# Machine Learning in Neuroimaging: A New Approach to Understand Acupuncture for Neuroplasticity

**DOI:** 10.1155/2020/8871712

**Published:** 2020-08-24

**Authors:** Tao Yin, Peihong Ma, Zilei Tian, Kunnan Xie, Zhaoxuan He, Ruirui Sun, Fang Zeng

**Affiliations:** ^1^Acupuncture and Tuina School/The Third Teaching Hospital, Chengdu University of Traditional Chinese Medicine, Chengdu, Sichuan, China; ^2^Acupuncture & Brain Science Research Center, Chengdu University of Traditional Chinese Medicine, Chengdu, Sichuan, China; ^3^Key Laboratory of Sichuan Province for Acupuncture and Chronobiology, Chengdu University of Traditional Chinese Medicine, Chengdu, Sichuan, China

## Abstract

The effects of acupuncture facilitating neural plasticity for treating diseases have been identified by clinical and experimental studies. In the last two decades, the application of neuroimaging techniques in acupuncture research provided visualized evidence for acupuncture promoting neuroplasticity. Recently, the integration of machine learning (ML) and neuroimaging techniques becomes a focus in neuroscience and brings a new and promising approach to understand the facilitation of acupuncture on neuroplasticity at the individual level. This review is aimed at providing an overview of this rapidly growing field by introducing the commonly used ML algorithms in neuroimaging studies briefly and analyzing the characteristics of the acupuncture studies based on ML and neuroimaging, so as to provide references for future research.

## 1. Introduction

Neuroplasticity usually refers to brain plasticity, which means the ability of the brain to modify its organization to the altered demands and environments [1, 2]. The cumulative evidence from both animal and human studies demonstrated that the adult mammalian brain was plastic and could be remodeled by the environmental input continuously [3–5]. The long-term noxious stimulus, such as pain and depression, as well as regular exogenous interventions can reorganize the structure and function of the brain [6–9]. As the most widely used complementary therapy, acupuncture is considered to treat diseases via facilitating neural plasticity from multiple pathways, such as promoting endogenous neurogenesis, modulating synaptic plasticity, and regulating the secretion of neurotrophins and neurotransmitters, so as to affect the structural and functional plasticity of the brain [10–13].

In the past two decades, studies on acupuncture promoting brain plasticity were greatly enhanced with the development of neuroimaging techniques. Several studies focused on investigating acupuncture-induced brain structural and functional plasticity by magnetic resonance imaging (MRI), positron emission tomography (PET), and other neuroimaging methods [14, 15]. People found that acupuncture could modulate the brain functional activities, shape the gray matter structure, and remodel the white matter fiber connection [16–18] and that the modulation of acupuncture on neuroplasticity varied with the different acupuncture modalities and different acupoint stimulations [19, 20]. For instance, our previous study [21] found that acupuncture could positively modulate the functional activity of the rostral ventromedial medulla in patients with migraine and that the neural plasticity elicited by puncturing at real acupoints was more pronounced than sham acupoints.

**Figure 1:**
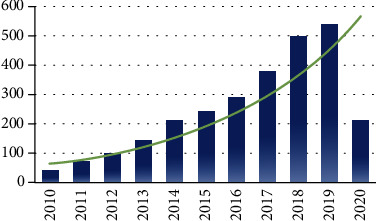
Numbers of publication on neuroimaging and machine learning in the last decade (from January 1, 2010, to June 1, 2020). The data was obtained by searching at the PubMed database with the items (Neuroimaging) AND (Machine Learning).

Currently, most neuroimaging findings of acupuncture facilitating neuroplasticity were obtained by the standard univariate analysis. It means the results were only significant at the group level, which limited their clinical translation to a certain extent. So, it is of great value to investigate how acupuncture promotes neuroplasticity and how the specific neuroplasticity affects the responses to acupuncture from the individual level. The application of multivariate pattern analysis (MVPA) and machine learning (ML) in neuroimaging studies provides an attractive method to this issue [22]. Since 2010, over 2200 studies focusing on ML in neuroimaging have been published in PubMed (pubmed. ncbi.nlm.nih.gov), and the number of these studies is increasing by 37% per year (Figure 1). With the ML algorithms and the neuroimaging features, researchers established the diagnostic and prognostic models of diseases. The interpretation of these models complemented the deficiencies of univariate analysis. They can not only assist in diagnosing diseases and in predicting individuals' responses to intervention but also provide novel insights for understanding brain plasticity. For example, Min et al. [23] found that schizophrenics who were sensitive to electroconvulsive therapy (ECT responders) had significantly higher whole-brain transfer entropy than the ECT nonresponders and that the value of whole-brain transfer entropy could be used as a reliable and plausible neuroimaging biomarker for random forest (RF) classifier to identify the ECT responders from the nonresponders. In another study, applying the baseline gray matter volume (GMV) of the subgenual cingulate cortex as a feature, Redlich and colleagues [24] successfully predicted the continued improvement of depression symptoms in patients with major depressive disorder following ECT. Simultaneously, integrating ML and neuroimaging technologies to investigate the facilitation of acupuncture on brain plasticity and using specific brain plasticity to predict acupuncture efficacy which can promote precision treatment have been a new focus in acupuncture research.

Therefore, we conducted this review by introducing the most widely used ML algorithms in neuroimaging studies briefly and analyzing these applications in the fieldof acupuncture promoting neural plasticity, aiming to provide an overview of this rapidly growing field and new approaches in future research.

## 2. Overview of Machine Learning in Neuroimaging

ML is a subfield of artificial intelligence which is aimed at investigating how computers can improve decisions and predictions based on data and ongoing experience [25, 26]. According to the criteria whether the training data is given a label or not, ML is divided into supervised learning, unsupervised learning, and semisupervised learning [27]. The unsupervised learning and semisupervised learning are generally applied for data reduction and feature selection [28], whereas the supervised learning is mainly used to construct the classification or regression models, which can learn the mappings between the input features and labels, to make individual-level estimations for the previously unseen data. The supervised learning includes many types, of which the most commonly used in neuroimaging research include support vector machine (SVM), decision tree (DT), RF, and artificial neural network (ANN) [29].

### 2.1. Support Vector Machine

The SVM is so far the most popular supervised learning algorithm in neuroimaging studies and is widely utilized in classification and prediction [30–33]. The principle of SVM is constructing a separating hyperplane that classifies all inputs, and the goal is searching for the optimal separating hyperplane that maximizes the margin between the hyperplane and the support vectors [34]. With different kernel functions, the distinct separating hyperplanes in different dimensions were constructed to perform the classification or prediction analysis. Among the different kinds of kernel functions in SVM models, the linear kernel and Gaussian kernel are most frequently used in neuroimaging studies [35–37]. The linear SVM is designed to solve the linear separating problems, while the RBF SVM is used primarily to seek nonlinear separating boundaries in the high-dimensional space.

### 2.2. Decision Tree and Random Forest

DT is the rooted directed tree that predicts the output based on a sequence of splits in the input feature space. The nodes split at each step by optimizing a metric, which indicates the consistency between the estimates and truth values. When the node has no subordinate to split, the traversal of this tree generates the target outcome prediction. As a typical classification algorithm with high interpretability, DT is applied predominantly for classification and disease diagnosis in neuroimaging studies [38, 39].

RF is generally the ensembles of DTs [40]. The principle of RF is consolidating multiple and diverse DTs together, and the final prediction outcome of RF is determined by the votes of each DT in the forest. As an integrated algorithm, RF can potentially yield much better prediction performance than learning with a single DT [41].

### 2.3. Artificial Neural Network

The concept of ANN is derived from the biological neural network. Similar to the synaptic connection in the brain, an ANN is composed of several layers of interconnected artificial neurons that make up the input layer, hidden layer, and output layer. As an ultracomplex ML algorithm, ANN establishes the computational units of multiple layers by simulating signal transmission and learning the architecture of synapse [42]. Due to the flexibility of its structure, ANN has the ability to fit arbitrarily complex functions given sufficient annotated data [27]. Traditionally, the utilization of ANN is extremely limited in neuroimaging for the small training samples, while in recent years, with benefit from the open-access of the large-scale neuroimaging data repositories, the application of ANN is accelerating and has great potential to become one of the most efficient algorithms in neuroimaging studies [43, 44].

**Figure 2:**
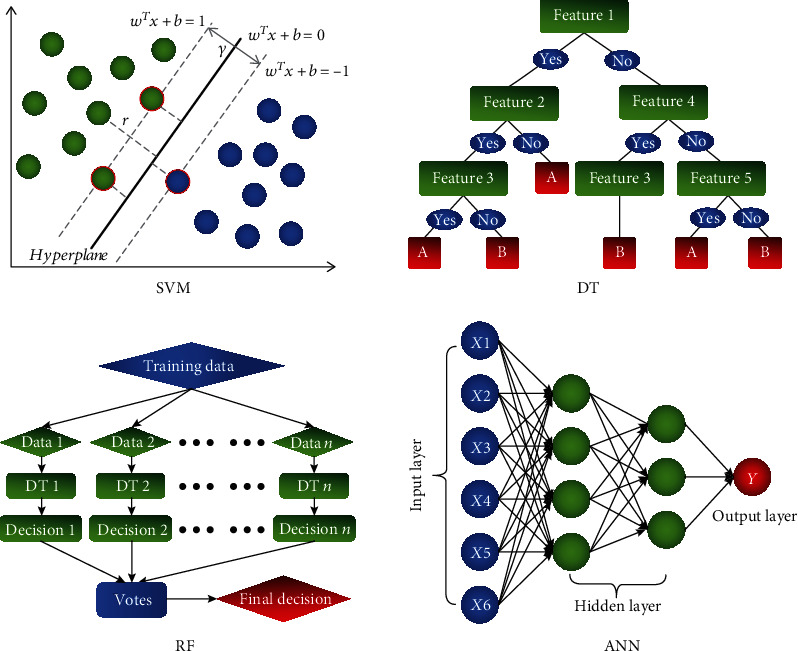
Diagrams of the commonly used machine learning algorithms in neuroimaging studies. SVM: support vector machine; DT: decision tree; RF: random forest; ANN: artificial neural network.

The diagrams of the above algorithms are summarized in Figure 2.

## 3. Application of Neuroimaging and Machine Learning in Acupuncture Promoting Neuroplasticity

In this review, we focused on the application of neuroimaging and ML in acupuncture promoting neuroplasticity. For a comprehensive summary of the field, we systematically searched papers in PubMed (pubmed.ncbi.nlm.nih.gov), Web of Science (https://www.webofknowledge.com), EBSCO (search.ebscohost.com), and CNKI (https://www.cnki.net) databases. According to the established inclusion and exclusion criteria, a total of ten studies were finally included [45–54]. The details of data acquisition and literature selection process can be found in the supplementary materials (available here).

These ten studies were published from 2008 to 2020. Generally, for participant selection, these studies were performed on healthy subjects [45–47, 49, 54], patients with migraine [48, 50, 51], patients with chronic low back pain [53], and patients with functional dyspepsia [52], respectively. The sample size of these studies ranged from 12 to 94, and the average sample size of healthy subjects was 28. Except for one study that enrolled participants with a wide age span [54], these studies mainly included participants aged 20-45 years. For acupuncture intervention, nine studies [45–53] applied manual acupuncture, and one study [54] selected the electroacupuncture as the intervention method. For scan design, six studies [45–49, 54] applied the on-off block design to detect the real-time effects of acupuncture on functional brain plasticity. For imaging parameters, seven studies [45–47, 50–53] employed MRI to acquire neuroimaging data and applied the blood oxygenation level-dependent (BOLD) signal [45–47], functional connectivity [52, 53], GMV [51], and diffusion measures of white matter fibers [50] to reflect the structural and functional patterns of the brain. For machine learning parameters, eight studies [45–52] were aimed at solving the problems of pattern classification, and the other two studies [53, 54] were designed to predict pain relief following acupuncture treatment. SVM, especially the linear SVM, was the most used ML algorithm in classification [45–52], whereas support vector regression [53] and fuzzy neural network [54] were applied in predictions. Three studies [49, 53, 54] exploited a hypothesis-based approach and selected the features of interest as inputs, and the other three studies [45, 50, 51] integrated multiple methods to find the optimal inputted features. Cross-validation, particularly the leave-one-out-cross-validation, was the popular validation strategy [45, 47–53], and only one study used independent samples as the validation set in these ten studies [54].

The detailed characteristics of these included studies were displayed in Table 1.

### 3.1. Concerns of Studies on Acupuncture Promoting Neuroplasticity

According to aims and design, these studies can be divided into three types. Among them, three studies [45–47] focused on the acupoint specificity, two studies [48, 49] were concerned with the differences and similarities of different acupuncture manipulations, and five studies [50–54] paid their attention to the prediction of acupuncture efficacy.

#### 3.1.1. The Acupoint Specificity

Acupoint specificity refers that acupoints have different therapeutic effects and biophysical characteristics compared to sham acupoints and that different acupoints have relatively different therapeutic effects and biophysical characteristics [55]. In this review, three studies [45–47] focused on the acupoint specificity. One study was aimed at exploring the differences in real-time brain functional plasticity elicited by a verum acupoint and a sham acupoint. The other two studies compared the differences between different acupoints (GB40 vs. KI3 [46] and HT7 vs. PC6 [47]). These three studies had similar experimental designs, including focusing on the different points in the same nerve segments, using the multiple on-off block design, choosing the BOLD signal as features, and adopting the linear SVM algorithm to build models. For example, Li et al. [45] applied MVPA and searchlight method to decode spatial discrimination of acupuncture stimulation at GB37 and a nearby sham acupoint. The results indicated that the occipital cortex, limbic-cerebellar areas, and somatosensory cortex were the main regions with higher classification accuracy in the discrimination of the verum and sham acupoint stimulation. These studies indicated that acupuncture stimulation at different points induced distinct real-time brain functional plasticity in different regions and that MVPA could be used to investigate the real-time neuroplasticity from the individual level. Interestingly, these three studies utilized the general linear model (GLM) analysis to verify the findings obtained in MVPA, while every GLM analysis showed that the different points caused similar BOLD signal changes. It suggested that the conventional univariate analysis might not be sensitive enough to detect the neural plasticity evoked by different acupoint stimulation. This is consistent with the opinion that multivariate analysis was more sensitive than univariate analysis in neuroimaging studies [56].

Acupoint specificity is not only the core of acupuncture theory and the base of clinical practice but also the focus of acupuncture-neuroimaging research [57–59]. Our previous review [15] indicated that more than 1/3 acupuncture-neuroimaging studies focused on acupoint specificity and these studies mainly concentrated on the differences of verum acupoints and sham acupoints. From 1995 to 2016, 79 original neuroimaging articles on acupoint specificity were published in PubMed, and 53 articles focused on the difference between the verum acupoints and the sham acupoints [19]. Given the importance of acupoint specificity in acupuncture theory and clinical practice and the extensiveness in neuroimaging research, we hold that acupoint specificity is bound to become a hot spot in future ML and neuroimaging studies.

#### 3.1.2. Acupuncture Manipulation

Two [48, 49] of the ten studies centered on the differences in brain functional plasticity caused by the different acupuncture manipulations. In one study [48], linear SVM was applied to classify the baseline and post acupuncture blood perfusion patterns in both verum and sham acupuncture groups. The results illustrated that the SVM classifier performed better when the training data was extracted from the verum acupuncture group. Moreover, the temporal lobe and cerebellum contributed important information for the discrimination in the verum acupuncture group. Another study [49] proposed a classification framework based on multiple ML algorithms for the two traditional acupuncture manipulations: the twirling-rotating manipulation and lifting-thrusting manipulation. The results demonstrated that with all the six graph theory properties as inputs, the SVM classifier got the highest accuracy of 92.14%. Moreover, the *post hoc* analysis also found the significant between-group differences of these six graph theory measures between two manipulations.

Acupuncture manipulation is the key in acupuncture clinical practice and significantly affects acupuncture efficacy [60]. In more than 2000 years of development, acupuncture has formed a rich variety of modalities and manipulation skills. The differences between acupuncture and moxibustion, electroacupuncture and manual acupuncture, acupuncture with *deqi* and acupuncture without *deqi*, and the reinforcing manipulation and reducing manipulation are always the key of clinical and experimental research in the acupuncture field and could be the research direction in future MVPA studies.

#### 3.1.3. Prediction of Acupuncture Efficacy

The integration of ML and neuroimaging features has been extensively employed in predicting the clinical efficacy of drugs or other interventions [33, 61]. In this review, five studies focused on acupuncture efficacy prediction [50–54]. Among them, three studies [50–52] adopted the classification algorithms to predict patients' responses to acupuncture treatment. For example, Liu et al. [50] utilized the diffusion measures of the medial prefrontal cortex- (mPFC-) amygdala fiber as inputs and established a linear SVM classifier to predict the response of migraine patients to the 8-week sham acupuncture treatment. The result showed that when using each single diffusion measure as input, the accuracy of the classifier is lackluster, whereas when multiple measures were applied the classifier could accurately discriminate responders from nonresponders with an accuracy of 84.0%. Moreover, the most discriminative white matter plasticity features that contributed to the classification were located in the external capsule, anterior cingulate gyrus, and mPFC. The other two studies [53, 54] constructed the regression models to predict the continuous improvement in symptoms after acupuncture treatment. For example, Tu et al. [53] used the features of interest as inputs to predict pain relief in patients with cLBP following 8-week verum or sham acupuncture treatment. The results showed that multiple functional connections involving mPFC could provide vital information for predicting the improvement of symptoms after both verum and sham acupuncture treatment.

These five studies on acupuncture efficacy prediction demonstrated that the specific neuroplasticity features including morphology of gray matter and white matter and cerebral functional activity patterns contained vital information for predicting the response of patients to acupuncture stimulation. The integration of ML and neuroimaging provides a new and promising approach for investigating mechanisms of acupuncture efficacy at the individual level, which has great potential for clinical translation and will be the important growth pole in acupuncture research.

In addition to the three aspects described above, there are still some other concerns that should be focused in future neuroimaging-based ML studies, for example, investigating the influences of acupuncture with different acupoint combination or different stimulation intensity on neural plasticity and predicting clinical efficacy of acupuncture with the neuroimaging features acquired under acupuncture stimulation.

### 3.2. Design of Machine Learning in Studies on Acupuncture Promoting Neuroplasticity

The application of neuroimaging techniques in acupuncture mechanism has produced remarkable advance[57, 62, 63] and developed a series of proven execution specifications [14, 19, 64]. In contrast, the integration of ML and neuroimaging in acupuncture research is still in its early stage, which inevitably brings many challenges but also the future directions.

#### 3.2.1. Sample Size

Due to difficulties in data acquisition, the sample size of neuroimaging study is generally small [65, 66]. By reviewing the studies which integrated ML and neuroimaging technologies to investigate neuropsychiatric disorders, Sakai and Yamada [29] found that 45.6% of the studies from 2014 to 2018 had a sample size of fewer than 100 cases. In our review, the sample size of the included studies ranged from 12 to 94 and six studies had a sample size of fewer than 50 cases. A small sample size exacerbates the possibility of adaptive models to learn noise, which leads to the high variability of estimates and overvaluation of prediction accuracy [67]. Simulation experiments showed that even when the sample size in the neuroimaging study reached 100 cases, the error bars were still around 10% [68]. Only when the samples of the training set exceeded 200 cases did the prediction model's performance begin to plateau [69]. Therefore, when conducting an ML study to predict the efficacy of acupuncture based on the neuroimaging properties, a sample size of 200 or more cases should be guaranteed whenever possible.

#### 3.2.2. The Appropriateness of Feature Selection

Considering that there are generally more features than samples in neuroimaging data, it is beneficial to take appropriate manners to eliminate the redundant features and reduce the dimension of data. The ten studies included in this review indicated that when using a single feature as input, the accuracy of the classifier is lackluster, whereas when multiple neuroimaging features applied, the accuracy of the model was significantly improved [49, 50]. This finding suggested that the properties of neuroplasticity that influenced the efficacy of acupuncture were multidimensional and complex. Moreover, another interesting finding was that both GMV and diffusion measures of white matter fiber could accurately discriminate between acupuncture-sensitive and acupuncture-insensitive migraine patients [50, 51]. Does it mean that the prediction model achieves better performance to discriminate the acupuncture responders and acupuncture nonresponders if both gray matter and white matter features are applied as inputs? In fact, the previous studies have illustrated that using multimodal rather than single-modal neuroimaging features as inputs can induce higher classification accuracy and better prediction performance [70, 71]. Therefore, future studies could attempt to use multimodal neuroimaging features as inputs to further explore the multidimensional features that predict the efficacy of acupuncture accurately.

#### 3.2.3. The Representativeness of Training Data

The current ML studies generally favor seeking homogeneous subjects to establish classification and prediction models [72–74]. It reduces the underfitting of the model caused by data heterogeneity, but severely limits the generalizability of the model to the real-world data [75]. The requirements for the representativeness of training data depend on the purpose of the study. For example, when a study is aimed at investigating the effects of different acupuncture manipulations on brain plasticity, the participants should be the homogeneous individuals from the same site. However, if the study is aimed at creating a generalizable model to predict the clinical efficacy of acupuncture, the participants should be enrolled from multiple centers to represent the heterogeneous population in real life.

#### 3.2.4. The Validity of Labels

The goal of ML is establishing mappings between training data and labels and then use the mappings as benchmarks for predicting the labels of the unseen data. Similar to other ML studies [76–78], the majority of current studies on acupuncture efficacy prediction use the subjective symptoms as the labels. These labels obtained with self-evaluated symptoms are subject to individual cognitive bias and have a high degree of variability. The heterogeneity yielded by subjective labels may hamper ML algorithms to discover optimal neuroimaging biomarkers and establish accurate mappings between data and labels. Therefore, applying objective biological markers as labels to establish an objective-to-objective mapping between features and labels should be taken into consideration in future studies to reduce the influence of subjective factors on model reliability.

## 4. Conclusion

In summary, we provided an overview of the literature on the application of ML and neuroimaging in acupuncture promoting neural plasticity. Studies published so far have preliminarily demonstrated at the individual level that different acupoint stimulation and different acupuncture manipulations had significantly different real-time modulatory effects on functional brain plasticity and that the specific structural and functional neuroplasticity features at baseline could accurately predict the improvement of symptoms following acupuncture treatment. Although this research field is currently in its early stage and faces many challenges, we still believe that integrating ML and neuroimaging techniques will be a promising approach to understand the facilitation of acupuncture on neuroplasticity in the future.

## Figures and Tables

**Table 1:** The detailed characteristics of the included studies.

	Participants Sample size, gender (M/F), age (Y)	Intervention	Modality	Feature	Purpose (C/R)	ML	Feature selection	Validation	Model assessment	MVPA findings	Univariate analysis results	Conclusion
López et al., 2013	Migraine Verum ACU: 18, 3/15, 44.44 ± 9.65; sham ACU: 18, 7/11, 43.94 ± 11.98	One session of verum or sham ACU stimulation	Task-SPECT (function) Task: ACU when image data acquisition	Blood perfusion	C	Linear SVM	Filter: discarding voxels with intensity values under 25% of the maximum	LOOCV	ACC SPE SEN	The classifier performed better when the training data was extracted from the verum ACU group than from the sham ACU group.	Verum ACU yielded greater changes in the perfusion patterns than sham ACU. Verum ACU produced a more significant decrease in blood perfusion.	SVM can distinguish the SPECT images of pre- and post-ACU acquisitions. Changes in blood perfusion following verum ACU is greater than sham ACU.
Jung et al., 2019	HS 14, 14/0, 22.1 ± 1.1	ACU at L HT7 or L PC6 for 20 blocks	Task-fMRI (function) Task: block design (16 s rest+6 s ACU+4 s stimulation location+4 s intensity report) ∗20	BOLD signal	C	Linear SVM	No feature selection steps	LOOCV	ACC	The classifier got an accuracy of 58.6% for classifying HT7 and PC6 with the features extracted from SI, MI, paraCL, anterior and posterior insula, SMG, ACG, vmPFC, PPC, and IPL. Using signal of ROI as feature, the classifier got higher accuracy (MI, 65%; SMA, 64%; SMG 62%; SI, 62%; and dlPFC, 62%).	No significant difference in BOLD signal alteration following HT7 and PC6 stimulation	Spatial localization of pain perceptions to ACU needle can be predicted by the neural response patterns in the somatosensory areas and the frontoparietal areas.
Yu et al., 2019	HS TR: 30, 16/14, 23-27; LT: 30, 18/12, 23-28	One session of TR or LT manipulation at ST36	Task-EEG (function) Task: ACU manipulation (TR or LT) for 3 min	Graph theory	C	DT NB SVM KNN LDA BP TSK	Selecting the features of interest	6-fold CV	ACC AUC	The classifier got an accuracy of 92.14% and AUC of 0.9570 with all graph theory features as inputs. With the increase of filter number, the accuracy was gradually improved. The highest accuracy was 92.37% with 6 filters in the TSK model.	PLV of TR was stronger than the baseline, while PLV of LT was weaker than the baseline. The value of all the six graph theory features of TR was significantly lower than that of LT.	Different ACU manipulations have different effects on functional brain networks. Classification of different ACU manipulations based on EEG with network features is feasible.
Liu et al., 2018	MWOA Responder: 38, /, 22.1 ± 0.45; nonresponder: 56, /, 21.5 ± 0.43	24 sessions of sham ACU at NAP in 8 weeks	DTI (structure)	TABA	C	Linear SVM	Filter+wrapper: traversing the *p* values of the two-sample *t* test from 0.01 to 1 with a 0.01 interval to find the best *p* for classifier	LOOCV	ACC SPE SEN PPV NPV	The single FA, MD, AD, and RD of the mPFC-amygdala fiber contributed to lackluster classification accuracy. The classifier got a higher accuracy with the combined features of FA, MD, and RD (in which ACC, SEN, SPE, PPV, and NPV were 84.0%, 90.2%, 76.7%, 82.1%, and 86.8%, respectively). The external capsule, ACG, and mPFC significantly contributed to the discrimination of responders and nonresponders.	The increased FA, decreased MD, decreased AD, and decreased RD of the mPFC-amygdala fiber were detected in MWOA patients than HS.	The variability of placebo treatment outcomes in migraineurs could be predicted from prior diffusion measures along the fiber pathways of the mPFC-amygdala.
Yang et al., 2020	MWOA Responder: 19, 6/13, 35.0 ± 10.4; nonresponder: 22, 2/20, 37.5 ± 11.87	12 sessions of ACU at GV20, GV24, bil-GB13, bil-GB8, and bil-GB20 in 4 weeks	T1 (structure)	GMV	C	Linear SVM	Filter+wrapper+embedded: traversing the *p* values of the two-sample *t* test from 0.0025 to 0.05 with a step of 0.0025 to select the best *p* for classifier LASSO	10-fold CV	ACC SPE SEN AUC DSC	Using the clusters located at the frontal, temporal, parietal, precuneus, and cuneus gyri as features, the classifier got the SEN of 73%, SPE of 85%, ACC of 83%, and AUC of 0.7871.	The baseline GMV in all predictive regions significantly differed between responders and nonresponders. Alterations of migraine days were correlated with the baseline GMV of L cuneus, R MiFG/IFG, L IPL, and SPL/IPL. The responders achieved an increase in GMV of the L cuneus after ACU.	The pretreatment brain structure could be a novel predictor for ACU treatment of MWOA.
Tu et al., 2019	cLBP Real ACU: 24, 8/16, 39.0 ± 12.6; sham ACU: 26, 11/15, 40.0 ± 13.7	6 sessions of ACU in 4 weeks, 8-12 effective acupoints were used in the real ACU group; 12 sham points were used in the sham ACU group.	Resting-fMRI (function)	ICA+rsFC	R	RBF SVR	Selecting the features of interest	5-fold CV	*R* ^2^ MAE	The prediction model obtained an *R*^2^ of 34.3 ± 5.5% and an MAE of 1.67 ± 0.02 between actual and predicted treatment responses for real ACU. mPFC FC (mPFC-insula, mPFC-putamen, mPFC-caudate, and mPFC-AG) and other FC (PCC-MiFG, insula-IFG, insula-SPL, and caudate-AG) significantly contributed to prediction. The prediction model got an *R*^2^ of 29.3 ± 5.3% and an MAE of 1.52 ± 0.04 for sham ACU. Connections of mPFC-dACG, mPFC-SPL, mPFC-paraCL, SFG-PreCG, SFG-MiFG, and ACG-paraCL provided significant information for prediction.	Changes of pain severity correlated with baseline mPFC-SN and mPFC-AG FC in the real ACU group. Baseline mPFC-dACG FC was correlated with changes in pain severity in the sham ACU group. Changes of FC between the mPFC and insula/AG were correlated with the relief of pain severity after real treatment, while changes of FC between the mPFC and paraCL/SPL were correlated with the relief of pain severity after sham ACU treatment.	Pretreatment rsFC could predict symptom changes for real and sham treatment, and the rsFC characteristics that were significantly predictive for real and sham treatment differed.
Xue et al., 2011	HS 12, 9/3, 21-26	ACU at GB40 or KI3 for 3 blocks, switching after a one-week interval	Task-fMRI (function) Task: on/off block design (1 min rest+1 min ACU) ∗3	BOLD signal	C	Linear SVM	Singular value decomposition	/	SDM	The performance of the classifier was not mentioned in this study. ACU stimulation at GB40 produced predominantly signal increases in the insula, red nucleus, thalamus, and amygdala. ACU at KI3 elicited more extensive decreased neural responses in the MFG, PCC, thalamus, and ACG.	ACU at GB40 and KI3 can both evoke similar widespread signal decreases in the limbic and subcortical structures.	Neural response patterns between ACU stimulation at GB40 and KI3 are distinct. Conventional GLM analysis is insensitive to detect neural activities evoked by ACU stimulation.
Yin et al., 2020	FD Responders: 21, 2/19, 21.52 ± 1.89; nonresponder: 12, 2/10, 22.83 ± 2.73	20 sessions of ACU in 4 weeks. One or two acupoints among CV12, ST36, and BL21 were used.	Resting-fMRI (function)	rsFC	C	Linear SVM	Wrapper: recursive feature elimination	LOOCV	ACC SPE SEN AUC	The classifier obtained an ACC of 84.9%, SEN of 78.6%, SPE of 89.5%, and AUC of 86.8%. The FC between R insula-L precuneus, L MiOFG-L thalamus, L insula-L ACG, R ACG-R temporal pole, R SOG-R cerebellum-3 contributed crucial information for prediction.	/	The whole-brain resting-state functional brain network has good predicting potential for ACU treatment to FD patients.
Hao et al., 2008	HS 60, /, 24-75	One session of electro-ACU at ST36	Task-EEG/ECG (function) Task: ACU when image data acquisition	BIS TPI LF/HF HR HRV	R	FNN	Selecting the features of interest	Validation with an independent set	AAE	With the FNN, the AAE of the estimation and true value is 10.2278.	/	The alteration of *β*-endorphin following electro-ACU can be predicted by monitoring EEG and ECG signal parameters.
Li et al., 2010	HS 11/11, 21.4 ± 1.8; GB37: 11, /, /; NAP: 11, /, /	ACU at GB37 or NAP for 2 blocks	Task-fMRI (function) Task: on/off block design (1 min rest+two 30 s ACU separated by a 50 s rest period+50 s rest)	BOLD signal	C	Linear SVM	Searchlight+singular value decomposition	LOOCV	ACC	The occipital cortex, limbic-cerebellar areas, and somatosensory cortex could help to differentiate the central neural response patterns induced by real or sham ACU stimulation with higher accuracy above the chance level.	Compared with the sham group, the ACU group induced higher signal intensity at some major regions of limbic-cerebellar system and small regions of the primary somatosensory cortex and supplementary motor area.	Neural response patterns of brain cortex to the ACU stimulation at GB37 and a nearby NAP could differ from each other effectively with the application of the MVPA approach.

M/F: male/female; Y: year; C/R: classification/regression; ML: machine learning; MVPA: multivariate pattern analysis; ACU: acupuncture; SPECT: single-photon emission computed tomography; SVM: support vector machine; LOOCV: leave-one-out-cross-validation; ACC: accuracy; SPE: specificity; SEN: sensitivity; HS: healthy subjects; fMRI: functional magnetic resonance imaging; BOLD: blood oxygenation level dependent; TR: twirling-rotating manipulation; LT: lifting-thrusting manipulation; EEG: electroencephalogram; DT: decision tree; NB: naïve Bayes; KNN: *K*-nearest neighbor; LDA: linear discriminant analysis; BP: BP neural network; TSK: Takagi-Sugeno-Kang fuzzy system; CV: cross-validation; AUC: area under the ROC curve; PLV: phase locking value; MWOA: migraine without aura; NAP: nonacupoint; DTI: diffusion tensor image; TABA: tractography atlas-based analysis; PPV: positive predictive value; NPV: negative predictive value; FA: fractional anisotropy; MD: mean diffusion; AD: axial diffusivity; RD: radial diffusivity; GMV: gray matter volume; LASSO: least absolute shrinkage and selection operator; DSC: dice similarity coefficient; cLBP: chronic low back pain; rsFC: resting-state functional connectivity; RBF: radical basis function; SVR: support vector regression; MAE: mean absolute error; SDM: spatial discriminance map; GLM: general linear model; FD: functional dyspepsia; BIS: bispectral index; TPI: tip perfusion index: LF/HF: low/high-frequency ratio; HR: heart rate; HRV: heart rate variability; FNN: fuzzy neural network; AAE: absolute average error; L: left; R: right; SI: primary somatosensory cortex; MI: primary motor cortex; paraCL: paracentral lobe; SMG: supramarginal gyrus; ACG: anterior cingulate gyrus; vmPFC: ventromedial prefrontal cortex; PPC: posterior parietal cortex; IPL: inferior parietal lobe; dlPFC: dorsolateral prefrontal cortex; mPFC: medial prefrontal cortex; MiFG: middle frontal gyrus; IFG: inferior frontal gyrus; SPL: superior parietal lobe; AG: angular gyrus; dACG: dorsal ACG; PreCG: precentral gyrus; SFG: superior frontal gyrus; SN: subcortical network; MFG: medial frontal gyrus; ITG: inferior temporal gyrus; MiOFG: middle orbitofrontal gyrus; SOG: superior occipital gyrus.

## Data Availability

There is no original data in this review.

## Supplementary Materials

Supplementary Materials The details of data acquisition and literature selection process.
