# Supplementary material for: Machine Learning in Neuroimaging: A New Approach to Understand Acupuncture for Neuroplasticity
Source: Neural Plast. 2020 Aug 24;2020:8871712. doi: 10.1155/2020/8871712 (PMC7463415; doi:10.1155/2020/8871712)
Supplement: Supplementary Materials — The details of data acquisition and literature selection process. [file 8871712.f1.docx]

**Data acquisition**

***Search strategy***

Electronic searches were conducted in PubMed, Web of Science, EBSCO, and China National Knowledge Infrastructure (CNKI) databases with language restricting to English and Chinese. Keywords related to neuroimaging, machine learning, and acupuncture were used as follows (**Table S1**). If available, MeSH terms were added for each search term in Medline, Web of Science, and EBSCO. After electronic searches, the snowballing search was employed to find additional eligible studies from the reference lists and bibliographies of the identified publications.

***Selection process***

The initial searching results obtained with the above strategies were uploaded to EndNote X9. After removing the duplicates and irrelevant records, two authors (TY and PM) screened the abstracts and full texts of the remaining records independently to determine whether these studies meet the appropriate inclusion criteria. Any uncertainty or disagreement between TY and PM was reconsidered by a third reviewer (ZT).

***Inclusion and exclusion criteria***

*Inclusion criteria*

Articles fulfilling the following criteria were included: 1) full-text studies published prior to June 01, 2020; 2) studies aimed to investigate the facilitation of acupuncture on neuroplasticity; 3) studies used manual acupuncture, electroacupuncture, and other peripheral stimuli which based on the traditional acupuncture theory as intervention; 4) studies applied Magnetic Resonance Imaging, Positron Emission Tomography, Electroencephalogram, and other neuroimaging techniques as research methods; 5) classification or prediction studies performed with machine learning methods.

*Exclusion criteria*

Articles meeting the following criteria were excluded: 1) studies undertaken with animals; 2) studies used transcutaneous electrical nerve stimulation, or transcranial magnetic stimulation as intervention; 3) conference abstracts, book, theses, narrative or systematic reviews, or meta-analyses.

***Data extraction***

The two independent reviewers (TY and PM) extracted data with a standard data extraction spreadsheet in parallel. Again, any inconsistency or disagreement was determined by ZT.

The following items were retrieved and extracted from every article:

1) Publication details: authors and year of publication.

2) Details of methodology: participants, intervention, imaging modality, and machine learning parameters (purpose, machine learning algorithms, feature selection, validation, and model assessment).

4) Results: multivariate pattern analysis findings and univariate analysis results.

5) Conclusion.

**Table S1** Details of search terms. The terms could be classified into three following categories: neuroimaging, machine learning, and acupuncture.

| **Machine learning** | **Neuroimaging** | | **Acupuncture** |
| --- | --- | --- | --- |
| - Machine Learning - Support Vector Machine - Deep Learning - Support Vector Regression - Multivariate Pattern Analysis - Decision Tree - Random Forest - Artificial Neural Network - Neural Network - 机器学习 - 支持向量机 - 深度学习 - 神经网络 - 多变量模式分析 | | - Neuroimaging - Magnetic Resonance Imaging - Functional Magnetic Resonance Imaging - Positron Emission Tomography - Diffusion Tensor Imaging - EEG - Magnetoencephalography - Brain Imaging - 神经影像 - 磁共振成像 - 脑电图 - 脑磁图 | - Acupuncture - Electroacupuncture - Acupuncture Therapy - Acupoint - 针灸 - 针刺 |

**Process of literature selection**

One hundred and eight records were found from all databases. After removing duplicates and conference abstracts, the remaining 72 records were independently reviewed by two reviewers for eligibility. According to the established inclusion and exclusion criteria, 46 records were excluded due to no machine learning. Fifteen records were excluded due to no acupuncture intervention, and two records were excluded due to no neuroimaging involvement. After the screening phase, the reference lists of these nine eligible articles were checked, and one additional record was supplemented. Finally, ten studies were included in this systematic review. The process of literature selection was shown at the **Fig. S1**.


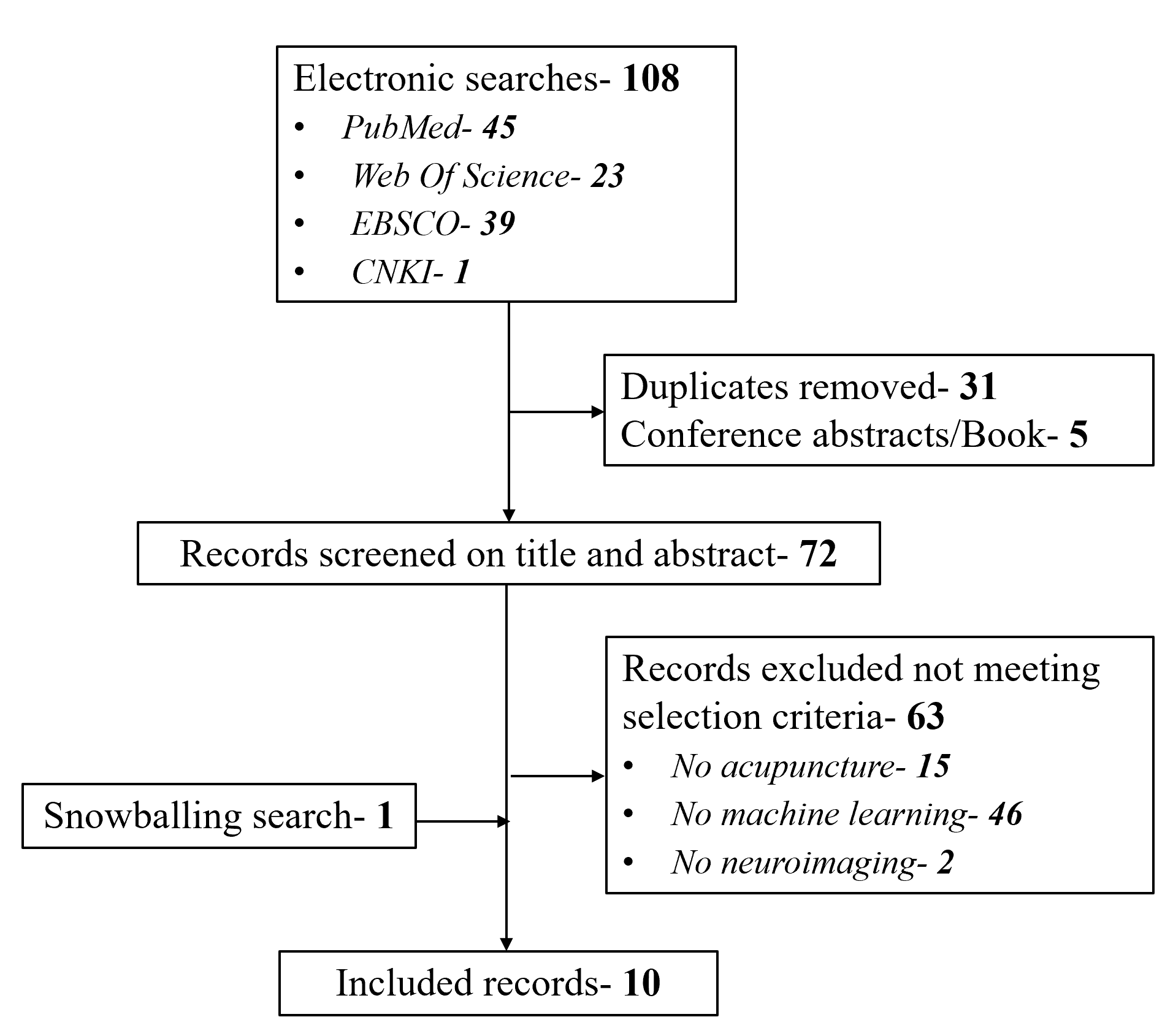


**Fig. S1** Process of literature selection.
